# Cell Death and Ageing – A Question of Cell Type

**DOI:** 10.1100/tsw.2002.163

**Published:** 2002-04-09

**Authors:** Pidder Jansen-Dürr

**Affiliations:** Institute for Biomedical Ageing Research, Rennweg 10, 6020 Innsbruck, Austria

**Keywords:** ageing, senescence, apoptosis, fibroblasts, endothelial cell

## Abstract

Replicative senescence of human cells in primary culture is a widely accepted model for studying the molecular mechanisms of human ageing. The standard model used for studying human ageing consists of fibroblasts explanted from the skin and grown into *in vitro* senescence. From this model, we have learned much about molecular mechanisms underlying the human ageing process; however, the model presents clear limitations. In particular, a long-standing dogma holds that replicative senescence involves resistance to apoptosis, a belief that has led to considerable confusion concerning the role of apoptosis during human ageing. While there are data suggesting that apoptotic cell death plays a key role for ageing *in vitro* and in the pathogenesis of various age-associated diseases, this is not reflected in the current literature on *in vitro* senescence. In this article, I summarize key findings concerning the relationship between apoptosis and ageing *in vivo* and also review the literature concerning the role of apoptosis during in vitro senescence. Recent experimental findings, summarized in this article, suggest that apoptotic cell death (and probably other forms of cell death) are important features of the ageing process that can also be recapitulated in tissue culture systems to some extent. Another important lesson to learn from these studies is that mechanisms of *in vivo* senescence differ considerably between various histotypes.

## REPLICATIVE SENESCENCE – A MODEL FOR HUMAN AGEING?

Human ageing is accompanied by a degeneration of various tissues that lose part of their physiological functions. Tissue degeneration is often accompanied by the loss of specialized cell types. In some cases, this is due to an exhaustion of the cell-division capacity, as is best documented for ageing of the immune system (for review, see [1]), and for part of the skin ageing program (for review, see [2]). At the cellular level, ageing phenomena can be reproduced *in vitro*

to some extent for cells derived from various tissues. The most prominent example is human diploid fibroblasts, which display a limited proliferation potential *in vitro* before they enter a stable growth arrest, defined as replicative senescence (for review, see [3]). Similar results have been described for primary human keratinocytes[4]. Moreover, replicative senescence was also observed in human T lymphocytes[1], human osteoblasts[5], and human endothelial cells[6], although in these cases, details of the senescent phenotype are less clear.

It has been shown that human fibroblasts arrest in the G1 phase of the cell cycle upon entering the senescent state (for review, see [7]), suggesting that deregulation of cell cycle progression may contribute to the senescent phenotype. Progression through the cell cycle is controlled by a family of cyclin-dependent kinases (cdks), heterodimeric enzymes that consist of a catalytic subunit and a specific cyclin that serves as a regulatory subunit[8]. The activity of cdks is controlled through cellular inhibitor proteins, referred to as cdkis (cyclin-dependent kinase inhibitors), which block the catalytic activity of specific cdks through physical association (for review, see [9]). It was shown that the abundance of the cdks p21(WAF1)[10], p16(INK4A)[11,12], and p27(KIP1)[12a] is strongly increased in senescent fibroblasts, which results in the inhibition of cdks[7].

While the initial studies of replicative senescence were focussed on the fact that senescent cells stop to proliferate, more recently there has been a shift of paradigm. There is now increasing evidence that age-associated changes in cellular differentiation and cell physiology contribute significantly to the loss of organ function. In this scenario, the appearance of a few senescent cells in a given tissue is a key determinant of age-related changes of tissue function. This is best illustrated with the example of human skin fibroblasts, which were shown to produce less collagen and to secrete more matrix-degrading enzymes during ageing *in vitro* (for review, see [2]). This fits well with the observation that the skin of aged individuals is characterized by a decreased collagen content, leading to the gross changes in skin morphology known as wrinkles. Moreover, it was also shown that senescent fibroblasts secrete large amounts of growth factors that support the growth of adjacent preneoplastic cells[13]. In studies of human skin biopsies, an age-related increase of senescent cells was observed *in vivo*[14], which may account for the age-related changes in skin morphology as well as the so-far unexplained age-related increases in certain types of skin cancer.

It has been hypothesized that oxidative stress is a major determinant of human ageing *in vivo*, and there is increasing evidence that this aspect of the ageing process can be reproduced *in vitro*. Thus, it has been shown that increasing the concentration of reactive oxygen species (ROS), for example, by increasing the oxygen partial pressure[15] or by treating cells with $H_2O_2$[16], can induce premature senescence in young diploid human fibroblasts. $H_2O_2$-treated human fibroblasts display cell-cycle arrest, senescence-associated β-galactosidase (SA βgal) staining, and other features of spontaneously senescent cells[17,18]. While these data suggest that oxidative stress contributes to the *in vitro* senescence phenotype of human fibroblasts, the role of reactive oxygen species for the ageing of other human cell types remains to be clarified.

## THE ROLE OF PROGRAMMED CELL DEATH IN HUMAN AGEING

Apoptotic cell death apparently plays an important role during ageing of various tissues *in vivo* (for review, see [19]), and it was shown recently that mice with a genetic defect in stress-related apoptosis display a significantly induced lifespan[20]. Tissue damage caused by age-dependent apoptosis has been documented in experimental animals for the brain[21], the inner ear[22], and the corpus luteum[23]. Similarly, it has been suggested that age-related apoptosis of T lymphocytes is an important determinant of immunosenescence in humans (for review, see [24]). On the other hand, it is assumed that apoptosis plays an important role in tissue homeostasis, and the failure of cells to exert the apoptotic program can also lead to disorders that accumulate during ageing. For example, it was suggested that decreased efficiency of apoptosis contributes to the alterations characteristic of intrinsic (chronologic ageing) and extrinsic (photoageing) skin ageing[25]. Together, these results suggest that regulation of programmed cell death plays an important role for the ageing process *in vivo*; however, the role of apoptosis for ageing may differ among various tissues.

Programmed cell death (apoptosis) can be triggered by a wide variety of environmental stimuli, and the apoptotic response of a given cell is modified by a plethora of cellular gene products (see box for details). At the cellular level, the issue of age-associated apoptosis is controversial. On the one hand, it was described that human T cells isolated from elderly persons display increased apoptosis[26], and apoptosis occurs *in vitro* after prolonged cultivation of CD4+ T cells[27]. On the other hand, the development of apoptosis resistance is also discussed as a key mechanism of immunosenescence[28], and senescent fibroblasts, which display no significant spontaneous apoptosis, are even resistant to proapoptotic signals[29]. Similarly, no increase in the rate of apoptosis was detected during *in vitro* ageing of human keratinocytes[30]. A recent study further explains the phenomenon of apoptosis resistance in senescent fibroblasts. Seluanov et al.[31] demonstrated that senescent human fibroblasts are indeed resistant to apoptosis, when apoptosis is induced with agents known to activate p53. In that study, the inability of senescent fibroblasts to undergo p53-dependent apoptosis was due to the inability of senescent fibroblasts to activate pathways that lead to p53 stabilization. When senescent fibroblasts are treated with agents that normally would induce p53, such as actinomycinD or UV irradiation, this causes a nonapoptotic type of cell death, namely necrosis[31]. However, p53-independent signals are well capable to induce apoptosis in senescent fibroblasts[31].

For ageing of the vascular system, a tissue-damaging role of apoptosis is well established[32]. Arteriosclerosis, a major age-related disease of humans, is accompanied by a degeneration of vascular endothelial cells and vascular smooth muscle cells[33] due to programmed cell death. In this context, the activation of the cellular suicide pathway leading to apoptosis of the endothelial cells represents an initial step in the development of arteriosclerotic lesions[34]. Increased incidence of apoptosis *in vitro* was also observed when vascular smooth muscle cells from human atherosclerotic plaques were grown *in vitro* and compared to control cells[35]. Whereas there is compelling evidence that cell death by apoptosis contributes to vascular damage under pathological conditions (e.g., arteriosclerosis), the role of apoptosis during normal vascular ageing remains to be established. Our finding that endothelial cells undergo age-associated apoptosis during *in vitro* ageing ([36]; see below) may provide a good model system to study this further.

## AGE-ASSOCIATED APOPTOSIS OF ENDOTHELIAL CELLS

To establish a system for studying vascular ageing at the molecular level, we have grown human umbilical vein endothelial cells (HUVEC) into replicative senescence and compared these cells to the standard fibroblast model of senescence. We found that, unlike senescent fibroblasts that enter a stable G1-arrest phenotype, senescent endothelial cells become polyploid and undergo significant programmed cell death. In senescent cells of both cell types, we found a similar accumulation of G1 cyclins and the cdkis p16, p21, and p27. The data suggest that the mechanisms leading to G1 arrest are probably shared between fibroblasts and endothelial cells; however, there are clearly profound cell type-specific differences in the senescence program with respect to the regulation of ploidy and apoptosis[36]. Interestingly, about 5% of the cells in the senescent HUVEC population stain positive for cyclin A at a given time point. It was shown that cyclin A gene expression is strictly dependent on activation of cyclin-dependent kinase 2 (cdk2)[37], suggesting that cdk2 remains active in a subpopulation of the senescent HUVEC[36]. Dysregulation of cdk activity was implicated in both apoptosis induction[38] and polyploidization[39,40,41]. Hence, the failure of senescent HUVEC cultures to completely downregulate cdks may explain both the proneness to apoptosis and the occurrence of polyploid cells. In a model derived from these findings, the low percentage of S-phase cells in a senescent HUVEC population could give rise to apoptotic cells. Age-related apoptosis is believed to play an important role during tissue ageing[21,22,23], and *in vitro* senescence of HUVEC may provide a new model for age-related apoptosis that could be useful for studying the role of intrinsic and extrinsic ageing in certain vascular pathologies, e.g., arteriosclerosis, which is characterized by excessive apoptosis[34].

**Box 1: Overview of apoptosis regulation in mammalian cells**

While much remains to be learned about apoptotic signal transduction and apoptosis execution, the current knowledge can be briefly summarized as follows. Apoptosis triggered by cell death receptors (e.g., the fas/Fas ligand system[42]) leads to the activation of a class of proteases, referred to as caspases[43], in particular caspase 8 and 10, which activate effector caspases (e.g., caspases 3, 6 or 7)[44]. Effector caspases then cleave key substrates and thereby cause nuclear fragmentation. In an alternative pathway, mitochondrial function is altered through a variety of signals, ultimately leading to the opening of the mitochondrial permeability transition pore (PTP). PTP opening is modulated, among others, by members of the Bcl-2 gene family (for review, see [45]). Once the PTP is open, soluble factors are released from the mitochondrial matrix that trigger, in one way or the other, an apoptotic response. These factors include cytochrome C which, in combination with the cellular protein Apaf-1[46], leads to activation of caspase 9. Caspase 9 in turn activates effector caspases (see above) and thereby results in cell death[47]. On the other hand, additional factors are released upon PTP opening. There is increasing evidence that mitochondrial death effectors do not necessarily activate caspases, and not all of these factors have been characterized. Recently, apoptosis inducing factor (AIF)[48] and endonuclease G[49] were shown to function as apoptotic effectors that are released from mitochondria and trigger caspase-independent cell death.

## BIOSKETCH

**Pidder Jansen-Dürr** is the head of the Department of Molecular and Cellular Biology of the Institute for Biomedical Ageing Research of the Austrian Academy of Sciences, Innsbruck, Austria. Dr. Pidder Jansen-Dürr has studied Biology and holds a Ph.D. in Zoology. His research interests include cellular ageing, cell cycle control, apoptosis regulation, viral oncogenesis, and metabolic control.
